# Intercellular Communication by Exosome-Derived microRNAs in Cancer

**DOI:** 10.3390/ijms140714240

**Published:** 2013-07-09

**Authors:** Bethany N. Hannafon, Wei-Qun Ding

**Affiliations:** Department of Pathology, University of Oklahoma Health Sciences Center, Oklahoma City, OK 73104, USA; E-Mails: bethany-hannafon@ouhsc.edu (B.N.H.); weiqun-ding@ouhsc.edu (W-Q.D.); Tel.: +1-405-271-3828 (B.N.H.); +1-405-271-1605 (W.-Q.D.); Fax: +1-405-271-3910 (W.-Q.D.)

**Keywords:** exosomes, microvesicles, microRNAs, cancer, breast cancer, microenvironment

## Abstract

The development of human cancers is a multistep process in which normal cells acquire characteristics that ultimately lead to their conversion into cancer cells. Many obstacles must be overcome for this process to occur; of these obstacles, is the ability to survive an inhospitable microenvironment. It is recognized that the intercommunication between tumor cells and their surrounding microenvironment is essential to overcoming this obstacle and for the tumor to progress, metastasize and establish itself at distant sites. Exosomes are membrane-derived vesicles that have recently been recognized as important mediators of intercellular communication, as they carry lipids, proteins, mRNAs and microRNAs that can be transferred to a recipient cell via fusion of the exosome with the target cell membrane. In the context of cancer cells, this process entails the transfer of cancer-promoting cellular contents to surrounding cells within the tumor microenvironment or into the circulation to act at distant sites, thereby enabling cancer progression. In this process, the transfer of exosomal microRNAs to a recipient cell where they can regulate target gene expression is of particular interest, both in understanding the basic biology of cancer progression and for the development of therapeutic approaches. This review discusses the exosome-mediated intercellular communication via microRNAs within the tumor microenvironment in human cancers, with a particular focus on breast cancer exosomes.

## 1. Introduction

### 1.1. Historical Perspective

Most cell types are known to continually release soluble factors and to exfoliate membrane derived vesicles into the extracellular space, including mast cells, dendritic cells, B-lymphocytes, platelets, neurons, adipocytes, endothelial cells and epithelial cells [1]. These membrane-derived vesicles are generally discriminated by size with two major classes; the larger class is called microvesicles (200–1000 nm) and the smaller class of nanometer size vesicles is called exosomes (30–200 nm). It is important to note that these are distinctly different from apoptotic bodies (0.5–3 μm), which are released from cells undergoing apoptosis or mechanical stress and are of a different cellular origin and molecular composition (see, for review, [2]). Exosomes were first observed three decades ago in differentiating reticulocytes. It was shown that during reticulocyte maturation, the transferrin receptor and many membrane-associated proteins were shed in small membrane vesicles via an unknown secretory process [3,4]. This process was considered as a way for cells to eliminate unwanted proteins and molecules, with exosomes functioning as cellular garbage disposals. However, in recent years, exosomes have emerged as important mediators of cellular communication that are involved in both normal physiological processes, such as lactation [4], immune response [5] and neuronal function [4], and also in the development and progression of diseases, such as liver disease [6], neurodegenerative diseases [7] and cancer. Exosomes have been identified in most bodily fluids, including urine and amniotic fluid [8], serum [9], saliva [10], breast-milk [5], cerebrospinal fluid [11], and nasal secretions [12]. Importantly, cancer cells have been shown to secrete exosomes in greater amounts than normal cells [13], indicating their potential use as biomarkers for diagnosis of disease.

### 1.2. Exosome Biogenesis and Secretion

Although the detailed mechanism for exosome biogenesis remains incompletely defined, current models suggest that exosomes are formed within the endocytic pathway and released from the plasma membrane via multivesicular bodies (MVBs) [14]. MVBs are formed during the maturation of early endosomes into late endosomes with the accumulation of intraluminal vesicles (ILVs), which correspond to exosomes [15]. Upon maturation, MVBs are either destined for fusion with the lysosome, where their contents will undergo lysosomal degradation, or with the plasma membrane, where their contents are released into the extracellular space. How these vesicles are sorted for either destination is not well understood. However, central players in this process are thought to be the endosomal sorting complexes required for transport (ESCRT). The ESCRT machinery is made up of five distinct complexes (ESCRT-0, -I, -II, -III and Vsp4; reviewed in [16]). This process is best characterized in yeast [17], where it was shown that the ESCRT machinery is responsible for generating MVB vesicles by initiating the budding of the endosome away from the cytoplasm and scission of the membrane to release of the mature MVB vesicles into the lumen of the lysosome (reviewed in [18]). ESCRT-0, -I and -II complexes recognize ubiquitinated proteins in the endosomal membrane [19], whereas the ESCRT-III complex may be responsible for membrane budding and vesicle scission [20]. However, there are additional pathways for MVB formation, sorting and exosome secretion. Most recently, an ESCRT-independent mechanism was described involving the sphingolipid, ceramide. Ceramide is generated during cellular stress and apoptosis either by *de novo* synthesis or by sphingomyelinase, the enzyme that hydrolyzes sphingomyelin into ceramide. Ceramide contributes to cellular signaling by playing a role in membrane microdomain coalescence, receptor clustering, vesicle formation, membrane fusion/fission and vesicular trafficking [21]. Additionally, ceramide is enriched in exosome membranes (see, for review, [21,22]). Further validating the ESCRT-independent process, inhibition of neutral sphingomyelinase (nSMase) decreased exosome formation and release, whereas depletion of different ESCRT components did not reduce exosome secretion or the formation of MVBs [23]. Interestingly, exosomes produced by the ESCRT-independent/sphingomyelinase pathway are enriched in tetraspanins, which are transmembrane proteins that may also be involved in endosomal sorting pathways [24]. Based on these observations, ESCRT-dependent sorting mechanisms may target proteins loaded into ILVs for lysosomal degradation, whereas ESCRT-independent sorting mechanisms may target ILVs for secretion. However, it is likely that this process is much more complex and may depend on the cell type, cargo or other stimulus. Furthermore, the signals that may control the switch between the two mechanisms remain unknown.

Exosome secretion is not considered a random event, but rather, a highly controlled process. Control of exosome secretion or “exocytosis”, although largely still under investigation, is thought to be coordinated through the transport and fusion of MVBs with the plasma membrane by the microtubule and actin cytoskeleton, t- and v-SNAREs and Rab GTPases [25]. Rab GTPases are ubiquitously expressed proteins that are responsible for the coordination of various vesicle trafficking events [26]. For example, overexpression of Rab11 has been shown to stimulate exocytosis [27], and Rab27a and Rab27b control different steps of the exosome secretion pathway [28]. Exosome secretion can be initiated by many different mechanical, chemical and biological stimuli. For example, DNA damage due to γ-irradiation activates the p53 tumor suppressor gene and induces the release of exosomes [29]. When breast cancer cells were cultured under hypoxic conditions (1% to 0.1% O_2_), their exosome secretion was significantly enhanced, whereas siRNA knockdown of HIF-1α prior to hypoxic exposure prevented this increase in exosome secretion [30]. Heparanase, an enzyme that cleaves heparan sulfate, which is upregulated in many cancers and is associated with enhanced tumor growth, was shown to dramatically increase exosome secretion in several human cancer cell lines [31]. Interestingly, heparanase could also alter the protein cargo carried by these exosomes, with increases in levels of syndecan-1, VEGF and HGF. Mechanical changes can also affect exosome secretion. For example, it was recently shown that detachment of adherent breast cancer cells from various surfaces could induce rapid exosome secretion [32]. Treatment with chemicals, such as calcium ionophores or statins to reduce membrane cholesterol levels and cholesterol biosynthesis, can also stimulate the release of exosomes in many cell types [23,33,34]. Finally, low pH level, a common hallmark of malignancy, in melanoma cells has been shown to increase exosome release and uptake, and pre-treatment with a proton pump inhibitor led to an inhibition of exosome uptake [35].

In order for secreted exosomes to exert any biological function, they must be absorbed by and deliver their contents to a recipient target cell. However, the specific targeting of exosomes to target cells and how this process unfolds in normal physiology or in the diseased state is not well understood. This process must critically depend on the specific adhesion molecules, integrins and antigenic factors expressed on the exosome, as well as the receptors or other docking molecules found on the surface of target cells. Presumably, any cell capable of endocytosis or phagocytosis may participate in the uptake of exosomes. Many studies have documented the uptake of exosomes by target cells; however, to date, only a handful of examples have described specific exosome and target-cell interactions. For example, exosomes from T-, B- and dendritic immune cells were shown to communicate with antigen presenting cells by transferring their contents in a unidirectional manner and modulating gene expression in the recipient cell [36]. Uptake of ovarian cancer secreted exosomes by NK cells has been demonstrated and was found to require the surface expression of phosphatidylserine (PS) as an uptake signal [37]. Lastly, the expression of galectin-5 on the surface of rat reticulocyte exosomes was also found to modulate their uptake by macrophages [38]. These studies have demonstrated that there are distinct signals that mediate exosome and target-cell interactions; however, more work is required to fully understand the distinct mechanisms controlling this process.

### 1.3. Exosome Components

According to the current version of the exosome content database, Exocarta (Version 4; http://www.exocarta.org), 4,563 proteins, 194 lipids, 1,639 mRNAs and 764 microRNAs have been identified in exosomes of many different cell types and from multiple organisms [39,40], thus demonstrating their complexity. The most frequently identified proteins in exosomes (as compiled by Exocarta) include membrane transport and fusion proteins, such as tetraspanins (CD9, CD63, CD81), heat-shock proteins (Hspa8, Hsp90), GTPases (EEF1A1, EEF2), and MVB biogenesis proteins (Alix). Other identified proteins include cytoskeletal proteins (actin, syntenin, moesin), metabolic enzymes (GAPDH, LDHA, PGK1, aldolase, PKM), signal transduction proteins (annexin, 14-3-3ɛ, 14-3-3ξ) and the carrier protein, albumin. The specific protein composition will depend on the cell type or tissue source from which the exosome originates and may fluctuate according to physiological changes. In addition, many of these proteins may function as specific exosome markers, particularly the tetraspanins, CD63 and CD81.

Beyond proteins, exosomes are also enriched in lipids and may act as cell-to-cell lipid mediators. Exosomes predominantly contain lipids, such as cholesterol, diglycerides, sphingolipids (including sphingomyelin and ceramide), phospholipids, glycerophospholipids (including phosphatidylcholine (PC), phosphatidylserine (PS), phosphatidylethanolamine (PE) and phosphatidylinositol (PI)) and polyglycerophospholipids (*i.e.*, bisphosphate). The ratio is increased for certain exosomal lipids when compared to parental cell lipids; these include sphingomyelin, PS, PC, PI and cholesterol, which can be present at as much as four times greater amounts and may account for the increased membrane rigidity of exosomes. Exosomes have also been reported to contain bioactive lipids, such as prostaglandins and leukotrienes, and active enzymes of lipid metabolism that may generate these lipids [13,41]. The presence of certain lipids, such as PS, on the outer membrane of exosomes can function in exosome recognition and internalization [42]. In this way, exosomes function as lipid carriers, allowing the transport of the bioactive lipids they carry to a recipient cell. This process of exosomal trafficking, particularly in the context of the tumor microenvironment, could lead to an enrichment of certain tumor progressive/immunosuppressive lipids, such as prostaglandins [43]. On the other hand, it may also lead to a replacement of harmful exosome lipid contents with beneficial ones. For example, docosahexaenoic acid (DHA), an omega-3 polyunsaturated fatty acid with many health and anticancer benefits, could be supplied by exosomes throughout the tumor microenvironment to affect cell-to-cell communication, reduce tumor cell growth and increase sensitivity to therapeutic interventions, particularly in breast tumors [44]. However, further studies are required to determine, which lipids participate in exosomal cell-to-cell communication and whether *ex vivo* manipulation is a plausible and/or effective therapeutic approach.

Exosomal transport of mRNAs and other non-coding RNAs, including microRNAs, was discovered only recently [45], and due to this exciting discovery, the interest in exosomes as carriers of genetic information is burgeoning, particularly in cancer research. Current reports have shown that the majority of the RNA present in exosomes is somewhat degraded and less than 200 nucleotides in length; however, recognizable proteins could be generated from *in vitro* translation of exosomal RNA extracts, thus demonstrating that full-length mRNAs are present [45]. Several studies have demonstrated that the RNA present in exosomes is very different from the parental cell RNA content, with the apparent lack of ribosomal RNA [36,45–47]. In contrast, the exosomal microRNA content is similar to that in the original tumor, thus peaking researchers’ interests in the use of exosomal microRNA profiles for cancer diagnostics [9,48]. However, an abundance of certain microRNAs that are not present or present at very low levels in the parental cells has recently been observed [49,50]. These results suggest that certain microRNAs may be preferentially secreted. However, the mechanisms for selective packaging and release of exosomal microRNAs are currently unknown, and whether these microRNAs may serve as reliable markers of disease is yet to be determined.

The first in-depth screening study was recently conducted to examine the entire transcriptome, miRNome and proteome of exosomes derived from melanoma cells and normal melanocytes [51]. Thousands of mRNAs that are associated with melanoma progression and metastasis, as well as several microRNAs (miR-31, miR-185 and miR-34b) that are involved in melanoma invasion were identified. In addition, several differentially expressed proteins, such as HAPLN1, GRP78, syntenin-1, annexin A1 and annexinA2, were identified, which were specific to the melanoma exosomes and may be involved in the malignant conversion of melanocytes. This study demonstrates the need for more in-depth explorations of exosome contents, so that specific targets may be identified and translated into clinical applications for disease biomarkers or potential therapeutic targets for cancer patients.

### 1.4. Exosome Isolation and Examination

Because exosome membranes are enriched in cholesterol, sphingomyelin, ceramide and lipid raft-associated proteins [52,53], they are highly stable and can be collected from various bodily fluids or from cell culture mediums [54]. Due to their small size and low density, exosome isolation usually involves multiple centrifugation and ultracentrifugation steps with a rotational force up to 100,000 × *g* for sedimentation. Centrifugation is also sometimes combined with 0.1 μm to 0.22 μm filtration in order to separate the nano-sized particles and to exclude larger particles and cellular debris, (see, for review of methods, [54]). For reduction of protein aggregate contamination and for obtaining a purer exosome preparation, sucrose, iodixanol [55], deuterium oxide density gradients (also called cushions) or proprietary reagents, such as ExoQuick (System Biosciences), have also be utilized [56,57]. Immunoaffinity capture methods can be used to isolate exosomes from cancer cells or patient serum using beads coated with antibodies against presumably any exosome-specific surface marker, such as the tetraspanins, CD63 or CD82, as a way to forego any ultracentrifugation. Epithelial cell adhesion molecule (Ep-CAM)-positive exosomes have been collected from the serum from lung [38] and ovarian cancer [48,58] patients, and HER2-positive exosomes have been isolated from HER2 overexpressing breast cancer cells [59] using this method.

Electron microscopy (EM) combined with negative staining is the standard method for visualization of whole-mount exosome preparations. Typical EM results show rounded vesicles with lipid bilayers, and sometimes, a “cup-shaped” morphology is observed, which may depend on the preparation process used. A review of these various isolation procedures and a comparison of the images obtained by EM analysis has been recently published [60].

Once the exosomes are collected, there are many downstream analysis options available. Exosomal proteins may be extracted utilizing standard cell lysis buffers or the TRIzol^®^ reagent (Life Technologies-Invitrogen) [61]. Proteins may be detected and analyzed by immunoblot procedures [61] or mass spectrometry (see, for review, [62]). Because the RNA content of exosomes is mostly small RNAs, the selection of RNA isolation technique is an important consideration. Various RNA extraction techniques, including phenol-based techniques (TRIzol^®^), silica column (e.g., RNeasy^®^ (Qiagen) or miRCURY™ (Exiqon)) and combined phenol and silica column approaches (e.g., TRIzol^®^ followed by RNeasy (Qiagen), miRNeasy (Qiagen) or mirVana™ (Ambion)) have been utilized and compared [63–65]. The RNA yield can be determined by spectrophotometric analysis at 260 nm, and a profile of the exosomal RNAs can be determined using the Agilent 2100 Bioanalyzer Lab-on-a-Chip instrument system (Agilent Technologies). Typical profiles of RNA extracted from exosomes contain a size distribution of 25–2000 nucleotides and are characteristically absent of ribosomal RNAs [63]. Detection of specific small RNA or microRNA species can be determined by real-time reverse-transcription PCR assay and oligonucleotide microarray analysis [51], or more in-depth analysis next-generation RNA sequencing can be applied [47,51,66].

Due to their nanometer size, the process of quantifying exosomes is somewhat of a challenge. There are two primary approaches currently used to quantify the amount of exosomes isolated from a preparation: quantification of the amount of exosomal protein using enzyme-linked immunosorbent assays (ELISA) or by immunoblotting. However, new approaches to quantify exosome secretion have been demonstrated using cell lines stably expressing GFP tagged CD63 (a specific marker of exosomes), thus generating exosomes with a traceable marker that can be easily measured by fluorescent spectrometry [32]. New nanoparticle/exosome tracking analysis technologies have recently been developed by Nanosight Ltd. [67]. These systems are equipped with a blue laser and camera that can visualize and measure nanoparticles within the 30 nm to 1,000 nm range (as demonstrated in [30,68]). Because the field of exosome research is in a phase of rapid growth, the refinement of isolation, imaging and visualization methods are expected to improve, along with the identification of specific molecular markers for isolation and the development of new technological approaches.

## 2. Exosomes and Intercellular Communication in Tumor Progression

### 2.1. Exosome Mediation of Intercellular Communication

It is well recognized that tumor development and progression is dependent on the reciprocal relationship between cancerous cells and their surrounding microenvironment. While the cancerous cells, which harbor many pro-tumorigenic genetic mutations, are the main driving force of tumor development, the surrounding stroma, which includes fibroblasts, endothelial and infiltrating immune cells, play a supportive and enabling role (reviewed in [69]). This reciprocal relationship requires not only a particular spatial interaction, but also the ability for the cancerous cells to communicate with the microenvironment by exchange of certain soluble proteins and genetic factors. Cancer cells are known to secrete factors that can promote the formation of new blood vessels, known as angiogenesis, to obtain oxygen to feed the tumor and to modify their adhesive properties in order to promote migration and invasion into the newly formed vasculature. Tumor cells of many different cancer types have been shown to secrete exosomes in greater amounts than normal cells [13], thus allowing the transfer of tumor-associated signaling molecules, including microRNAs, via fusion of the exosome with the target cell membrane [70].

Tumor-derived exosomes (TD-exosomes) are generally considered pro-tumorigenic. However, some anti-tumorigenic abilities have also been described. For example, exosome-like nanoparticles isolated from pancreatic cancer cells were shown to induce apoptosis in tumor cells [71]. Other studies have focused on the use of TD-exosomes as a source for tumor antigens for the development of exosome-based immunotherapies [72–74]. One report demonstrated that modified cell lines expressing interleukin-2 (IL-2) produced TD-exosomes containing IL-2 with increased antitumor effects [72]. A Chinese phase I clinical trial demonstrated that ascites-derived exosomes combined with granulocyte-macrophage colony stimulating factor could modulate the immune response and induce an antitumor cytotoxic T-lymphocyte (CTLs) response in 40 patients with colorectal cancer [73]. Another study showed that exosomes derived from IL-2 GPI-anchored renal cancer cells could induce CTLs and significant cytotoxic and antitumor effects *in vitro*, suggesting a novel strategy for an exosome-based vaccine for renal cell carcinoma [75]. Heat-stressed tumor cells were shown to produce exosomes that could attract and activate dendritic and T-cells, induce specific antitumor immune responses and inhibit tumor growth *in vivo* [76]. However, despite the above-described antitumor characteristics of exosomes, it is still unclear whether the immunomodulatory effects of exosomes secreted from tumor cells are either cancer-promoting or cancer-inhibiting, as no studies have demonstrated any immune stimulatory effects of TD-exosomes. Rather, many immune-evasion characteristics have been described. In this context, TD-exosomes from the ascites of ovarian cancer patients were recently shown to express the death ligands, FasL and TRAIL, which could trigger apoptosis in immune system cells, thereby inhibiting a tumor growth inhibitory immune response [77].

As described above, most initial studies on TD-exosomes were focused on their interaction with the immune system, while the effects of TD-exosomes on the tumor microenvironment has been less characterized. However, recent studies have shown that TD-exosomes have many pro-tumorigenic functions and are able to transfer their phenotypic traits (such as onco-proteins or onco-microRNAs) to a recipient cell and promote cancer stimulatory activities, such as proliferation, extracellular matrix remodeling, migration and invasion and angiogenesis, and contribute to the pre-metastatic niche formation for the promotion of metastasis. The following sections will discuss these topics in greater detail.

### 2.2. Exosome Modulation of Extracellular Matrix, Stromal Cells and Invasion

Remodeling of the extracellular matrix and alterations in cell-cell and cell-extracellular matrix interactions are the first barriers that must be overcome for a cancer cell to migrate and travel to distant sites. Several studies have shown that TD-exosomes can alter the extracellular matrix through secretion of matrix metalloproteinases (MMPs) or activators of MMPs, such as heat shock proteins. MMPs are zinc-dependent plasma membrane endo-peptidases that can degrade extracellular matrix proteins, such as collagen, fibronectin, proteoglycans and laminins. In fibrosarcoma and melanoma cells, it was shown that MT1-MMP was secreted in exosomes and could activate pro-MMP-2 and degrade collagen and gelatin [78]. Other studies have demonstrated that heat shock proteins, such as hsp90, are also secreted via exosomes and can activate MMP-2 to enhance invasion of cancer cells [79]. The role of platelets in tumor progression has been recently investigated, where it was found that platelet-derived microvesicles contribute to cancer progression in lung cancer cell lines by stimulating proliferation, cyclin D2 expression, adhesion to endothelial cells, invasion and angiogenesis through activation of mitogen-activated protein kinase (MAPK) p42/44, MT1-MMP, MMP-9, IL-8 and VEGF and other factors controlling these processes [80]. The effects of TD-exosomes on mesenchymal stem cells (MSCs) have also been explored. For example, TD-exosomes from ovarian cancer cells can induce a tumor-associated myofibroblast-like phenotype on adipose-derived MSCs, suggesting that exosomes contribute to the generation of tumor-associated fibroblasts in the tumor stroma [81]. Recently, Bobrie *et al*. showed that inhibition of Rab27a in breast cancer cells decreases the secretion of exosomes and MMP-9, resulting in a decrease in primary tumor growth and lung dissemination *in vivo*, thus further demonstrating that exosome secretion promotes tumor formation and progression and that inhibition of exosome secretion may impede tumor growth [82].

### 2.3. Exosome Stimulation of Tumor Angiogenesis and Metastatic Niche Formation

In order for a tumor to sustain its growth and survival, they have to obtain greater amounts of oxygen and nutrients through the formation of new blood vessels or by metastasizing to more hospitable organ sites. TD-exosomes have been shown to transport oncogenic and pro-angiogenic factors to cells within the tumor microenvironment to induce neoangiogenic activity and to promote premetastatic niche formation. For example, a hypoxic tumor microenvironment has been shown to enhance the secretion and transport of exosomes and pro-angiogenic protein factors that could potentially modulate the microenvironment to facilitate angiogenesis and metastasis [83]. Recently, *in vitro* hypoxia experiments in glioma cells and patient samples showed an enrichment of hypoxia-regulated mRNAs and proteins, such as MMPs, IL-8, platelet derived growth factor (PDGF), caveolin-1 and lysyl oxidase, in secreted exosomes. In addition, it was demonstrated that these exosomes are also potent inducers of angiogenesis through possessing these growth factors and cytokines [84]. Colorectal cancer cell-derived microvesicles/exosomes are enriched in cell cycle-related mRNAs that could promote proliferation of vascular endothelial cells [85] and in several metastatic and signal transduction molecules [86]. Microvesicles released from human renal cancer stem cells were shown to stimulate angiogenesis and promote the formation of a premetastatic niche in the lungs *in vivo* [87]. As mentioned above, MSCs can also promote tumor growth; for example, bone marrow MSC-derived exosomes can enhance VEGF expression in tumor cells by activating the ERK1/2 pathway [88]. Likewise, platelet-derived exosomes have also been shown to stimulate mRNA expression of angiogenic factors (such as MMP-9), as described above [80]. Exosomes released by chronic myeloid leukemia cells were shown to promote angiogenesis in a Src-dependent fashion [89]. Melanoma-derived exosomes were found to prepare bone marrow progenitor cells for a pro-metastatic phenotype through the receptor tyrosine kinase, MET [90]. A recent review proposes that the interaction and bidirectional exchange of genetic information, via secreted microvesicles, between macrophages and endothelial cells may work to promote vascular growth in the tumor microenvironment [91]. Overall, these studies clearly demonstrate that exosomes indeed function as pro-tumorigenic factors that can mediate intercellular communication in the tumor microenvironment and contribute to cancer progression.

## 3. Exosome-Derived microRNAs in Tumor Progression

### 3.1. Exosomes as Transporters of microRNAs

microRNAs are small (17–21 nt), non-coding RNAs that regulate gene expression at the post-transcriptional level through the RNA interference pathway. microRNAs are transcribed by RNA polymerase II as primary-microRNAs (pri-miRNAs) [92–94] and are processed in the nucleus by the enzyme, Drosha, into shorter hairpin structures of approximately 70 nucleotides in length, called pre-miRNAs. Pre-miRNAs are then transported from the nucleus, to the cytoplasm via Exportin 5 [95], where they are further processed into mature microRNA transcripts by the enzyme, Dicer [93]. The mature microRNA is then loaded into the ribonucleoprotein complex, known as the RNA induced silencing complex (RISC), and can bind in a sequence specific manner to the 3′ untranslated region (UTR) of target mRNAs, resulting in either translational inhibition or mRNA degradation [96]. Presently, ~2000 microRNAs have been described in humans [97] and a single microRNA may regulate many mRNAs; likewise, a single mRNA may be targeted by many microRNAs, establishing microRNAs as the largest class of gene regulators [98]. Through this mechanism, microRNAs are an essential component to regulating most cellular and developmental processes, including developmental timing, organ development, differentiation, proliferation, apoptosis and immune regulation (see, for review, [99]). Therefore, it is of no surprise that microRNAs are involved in cancer development and progression, and depending upon their target gene and level of expression, microRNAs may function as either tumor suppressors or oncogenes and assist in the promotion or suppression of cancer growth and progression [100]. Aberrant microRNA expression has been described across many cancer types, with global downregulation of microRNA expression seen as a common trend [101,102]. The transport of mRNAs and microRNAs by exosomes was realized only recently, but has led to an explosion of interest in cancer research. The first study to demonstrate exchange of nucleic acids via exosomes examined secreted exosomes from mouse and human mast cell lines [45]. Using standard RNA and DNA extraction techniques of exosomes isolated by ultracentrifugation, Valadi *et al.* discovered the presence of small RNAs and mRNAs (but not DNA) from approximately 1300 genes present in exosomes that are not present in the parental cell and proposed that these RNAs be referred to as exosomal shuttle RNAs (esRNAs) to distinguish them from circulating microRNAs [45]. In addition, these esRNAs could be *in vitro* translated into functional proteins and transferred to other human and mouse mast cells, where new proteins were generated in the recipient cells [45]. This seminal study has generated much interest in the study of cell-cell communication via delivery of small RNAs by transfer through exosomes.

### 3.2. Secretion and Uptake of Exosome-Derived microRNAs

The exact mechanism of microRNA loading into MVBs in the endocytic pathway and secretion via exosomes is not well understood. Studies thus far have demonstrated that MVBs are associated with GW-bodies, also known as P-bodies, which are cytoplasmic foci, where post-transcriptional regulation of mRNAs occurs, and are enriched in GW182 and AGO2 proteins, two main components of the RISC [103]. In this study, it was shown that endosomes or MVBs are sites of microRNAs, microRNA-repressible mRNAs and RISC accumulation and action and that exosomes secreted via MVBs are enriched in GW182, suggesting a mechanism for microRNA loading. Depletion of some of the ESCRT components compromised microRNA-mediated gene silencing and led to an over-accumulation of GW182, thus suggesting that GW182 and microRNA-loaded AGO2 are sorted in to MVBs via ESCRT components [103]. However, as previously mentioned, a more recent study has demonstrated that microRNAs are released through a ceramide-dependent secretory mechanism [104]. Furthermore, a tumor-suppressive microRNA, secreted via this mechanism, was taken up by a recipient cell, where it exerted gene silencing and growth inhibition [104]. The GW182 protein may also be important for microRNA stability and secretion via exosomes [105]. In this study, it was shown that knockdown of GW182 by siRNA increased microRNA instability and reduced secretion via exosomes, whereas replenishment of GW182 restored microRNA stability, thereby demonstrating a role of GW182 in protecting AGO2-bound microRNA [105].

Other studies have demonstrated that certain microRNAs are selectively secreted in exosomes. For example, the let-7 microRNA family is selectively secreted via exosomes in metastatic gastric cancer cell lines. Since this family of microRNAs targets oncogenes, such as Ras and HMGA2, they are generally considered a tumor-suppressive group of microRNAs; however, whether their release via exosomes is to promote or inhibit oncogenesis remains unclear [106]. Breast cancer cell lines selectively release the majority of miR-451 and miR-1246 via exosomes as compared to their parental cell, whereas these specific microRNAs are retained in non-malignant mammary epithelial cells and normal fibroblast cells [49]. The authors note that the biogenesis of miR-451 is dicer-independent [107], thus raising the possibility that non-canonical processing of microRNAs may target them for selective exosome release. Overall, these results suggest that there is a specific selection mechanism for microRNA release. However, the exact mechanism remains to be elucidated.

While the exact mechanism of exosome-derived microRNA uptake and processing in recipient cells is largely unknown, many types of cells have been shown to absorb exosomal microRNAs, where they can induce post-translational repression of target mRNAs. For example, T-cells have been shown to transfer microRNAs-loaded exosomes in an antigen-driven unidirectional manner to antigen presenting cells in the immune synapsis, where they modulate gene expression [36]. Additionally, mouse dendritic cells (DCs) release exosomes containing different microRNAs, depending on their level of maturation. These microRNAs were absorbed by recipient DCs, where they were shown to repress target mRNA expression [108]. One of the first studies to show that this unique intercellular method of communication could contribute to the initiation and progression of cancer demonstrated that hepatocellular carcinoma cells produce exosomes that can be internalized by other cells. These exosomes were shown to transmit microRNAs that modulated the expression of transforming growth factor β activated kinase-1 (TAK1), whose loss is implicated in hepatocarcinogenesis [109]. Thus, these studies have begun to shed light on the potential mechanisms of exosome uptake and the functional consequences of microRNA transfer.

### 3.3. microRNA Profiling of Tumor-Derived Exosomes in Clinical Samples

The discovery of the transport and exchange of microRNAs via exosomes has also generated much interest in the use of circulating TD-exosomes and their resident esRNAs as clinical diagnostic markers for cancer. To this end, a handful of studies have examined the microRNA profile from circulating TD-exosomes and compared the expression levels to the original tumor cells. For example, circulating TD-exosomes isolated from the serum of patients with ovarian cancer, age-matched controls and primary tumor cell cultures and matched sera were examined for microRNA expression changes. This study found that the expression of eight microRNAs, previously demonstrated to be diagnostic in ovarian cancer, were similar between cellular and exosomal microRNA preparations, thus suggesting that circulating TD-exosomes could be used as surrogate diagnostic markers for biopsy profiling, particularly in asymptomatic patient populations [9]. Another study from Taylor *et al*. evaluated the levels of microRNAs from the plasma of patients with lung adenocarcinoma, matched tumor samples and controls; they similarly observed no significant differences in exosome microRNA levels between microRNAs derived from circulating exosomes or from microRNAs from the primary tumors [48]. In another study, circulating exosomal/microvesicle-derived microRNAs were profiled from the plasma of prostate cancer patients with and without metastases [110], and a distinct set of 11 microRNAs was present at significantly greater amounts in patients with metastases compared to those without metastases. The association of two of these 11 microRNAs (miR-141 and miR-375) were confirmed in plasma exosomes from a separate patient cohort with recurrent or non-recurrent disease, thus demonstrating that changes in microRNA concentrations present in circulating exosomes from prostate cancer patients may be used for diagnosis and tumor staging [110]. In serum obtained from esophageal squamous cell carcinoma (ESCC) patients, microRNA expression profiling showed that miR-1246 was consistently elevated in patients *versus* controls and was an independent risk factor for poor survival [111]. The authors also indicate that miR-1246 was not upregulated in ESCC tissue samples; however, this observation is consistent with the previously mentioned report of preferential exosome secretion of miR-1246 from breast cancer cells [49]. Overall, these exosomal microRNA profiling studies, summarized in Table 1, have found that microRNA expression signatures are not significantly different between TD-exosomes and tumor cells, with the exception of miR-1246, suggesting that these circulating TD-exosome microRNAs could be utilized as a surrogate for biopsy microRNA profiling. In addition, a database called miRandola has been created to catalog all extracellular circulating microRNAs and currently contains 2312 entries with 581 unique mature microRNAs identified in circulation from 21 different types of samples [112].

### 3.4. Pro-Tumorigenic Effects of Exosome-Derived microRNAs *in Vitro*

**Figure 1:**
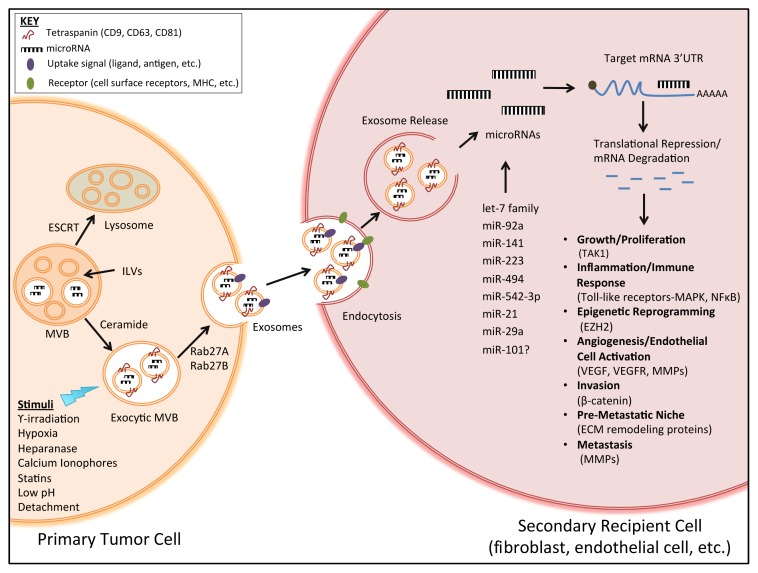
Biogenesis, secretion and uptake of tumor-derived exosomes in the tumor microenvironment. Exosomes are formed by the inward budding of the multivesicular body (MVB) membrane in the form of intraluminal vesicles (ILVs). Exosome formation and cargo sorting into lysosomes involves the endosomal sorting complex required for transport (ESCRT), which recognizes ubiquitinated proteins. Exosome production and secretion also occurs through an ESCRT-independent process involving the sphingolipid, ceramide, and the enzyme neutral, sphingomyelinase (the enzyme that converts sphingomyelin to ceramide). Exosomes secretion can be stimulated by various chemical, environmental and mechanical stimuli, such as gamma-irradiation, hypoxia (low oxygen), low pH, matrix detachment, *etc.* Exosomes are secreted in exocytic MVBs following fusion of MVBs with the cell membrane, a process that depends on Rab GTPases (Rab27A, Rab27B). Exosomes released from a primary tumor cell will display similar membrane components as their cell of origin, such as receptor ligands or antigens. Endocytosis of exosomes may occur through activation of cell surface receptors or bioactive lipid ligands. Upon endocytosis by a secondary recipient cell, such as fibroblasts or vascular endothelial cells, exosomes can release their microRNA cargo. The transferred microRNAs are functionally active and can regulate gene expression in the recipient cell through post-translational regulation of target mRNA expression, leading to mRNA degradation or de-stabilization. microRNA-dependent gene regulation can activate various processes involved in tumor development and progression. Abbreviations: TAK1, transforming growth factor β activated kinase-1; MMPs, matrix metalloproteinases; MAPK, mitogen activated protein kinase; NFκB, nuclear factor kappa-light-chain-enhancer of activated B-cells; EZH2, enhancer of zeste homolog 2; VEGF/VEGF, vascular endothelial growth factor/receptor; ECM, extracellular matrix.

The pro-tumorigenic effects of exosome-derived microRNAs after uptake by a recipient cell have recently begun to emerge. Thus far, exosome-derived microRNAs, through target gene transcriptional repression, have the demonstrated ability to induce cell migration, inflammation, immune responses, angiogenesis (including endothelial cell migration and tube formation), invasion, pre-metastatic niche formation and metastasis; see Figure 1. Therefore, these studies have implicated cancer cell exosome-derived microRNAs in most aspects of tumor progression. For example, leukemia cell exosomes have been shown to communicate with human umbilical vein endothelial cells (HUVECs), leading to increased cell migration and tube formation [113]. In this study, K562 leukemia cells were transfected with a Cy3-labeled pre-miR-92a and co-cultured with HUVECs. The Cy3-labeled miR-92a, derived from the K562 cells, could be detected in the cytoplasm of the endothelial cells and was co-localized with the exosomal marker, CD63. In addition, the expression of integrin α5, a target of miR-92a, was also greatly reduced in the recipient cells, thus demonstrating that an exosome-derived microRNA can function as an endogenous microRNA in a recipient cell and that exosomal microRNAs play an important role in cancer-to-endothelial cell communication [113]. As previously mentioned, microvesicles released from human renal cancer stem cells containing pro-angiogenic mRNA, and microRNA were shown to greatly stimulate endothelial cell growth and vessel formation and enhance lung metastases after *in vivo* implantation in a severe combined immunodeficient (SCID) mouse model [87]. In this study, molecular characterization of microvesicles, derived from CD105-positive (a mesenchymal stem cell marker) renal cancer stem cells, was conducted and was found to contain a set of pro-angiogenic mRNAs and microRNAs that are implicated in tumor progression and metastases [87]. The previously mentioned study, which profiled the miRNome of melanoma exosomes, identified 228 microRNAs that were differentially regulated in melanoma exosomes *versus* normal melanocyte exosomes, 15 of which are known to be associated with melanoma invasion and metastasis [51].

Tumor-associated macrophages, which are known to promote invasion and metastasis, have been shown to secrete microvesicles containing microRNAs that could be taken up by breast cancer cells. In a co-culture system, it was demonstrated that uptake of IL-4 activated macrophage secreted exosomes could promote the invasion of breast cancer cells, due to uptake of miR-223 (a microRNA specific for IL-4 activated macrophages) and disruption of the Mef2c-β-catenin pathway [114].

Through pathogen recognition receptors, such as Toll-like receptors (TLRs), and their associated downstream signaling pathways, such as nuclear factor kappaB (NF-κB) and MAPK, exosomal microRNAs may also play a large role in the regulation and homeostasis of the innate immune response by fine-tuning the mechanisms responsible for the production and release of cytokines/chemokines, adhesion and co-stimulatory molecules in epithelial cells (see, for review, [115]). These mechanisms, in the context of cancer, could be disrupted, thereby promoting an immune-evasion response and cancer promotion. For example, an interesting recent study has demonstrated that secreted microRNAs may act as ligands by binding to TLRs on recipient cells. Specifically, miR-21 and miR-29a secreted in exosomes from lung cancer cell lines were shown to bind to murine TLR7 and human TLR8 and triggered a TLR-mediated pro-metastatic inflammatory response that could lead to tumor growth and metastasis [116].

The process of malignant transformation may also alter the specific species of microRNAs that are secreted in exosomes or retained in cells. For instance, selective release of certain microRNA populations has been demonstrated in malignant breast cancer cells. Specifically, the microRNAs, miR-451 and miR-1246, produced by malignant breast epithelial cells are released, whereas the majority of these microRNAs are retained in non-malignant mammary epithelial cells [49]. A follow-up study from this same group demonstrated that these selectively exported microRNAs are packaged in exosomes that are larger than conventional exosomes and are enriched in CD44, a protein relevant to breast cancer metastasis. In contrast, they showed that normal cells release microRNAs in a homogenous type of vesicle, suggesting that the process of malignant transformation may alter the pathways by which microRNAs are exported from cells, thus leading to differences in exosome content and morphology [117].

TD-exosomes may also modulate pre-metastatic niche formation via long-range transfer of microRNAs. One study has demonstrated that exosomes from metastatic rat adenocarcinoma cells are preferentially taken up by lymph node stroma cells and lung fibroblasts [118]. The transferred microRNAs significantly affected mRNA translation of many genes, including proteases, adhesion molecules, chemokine ligands, cell cycle- and angiogenesis-promoting genes and oxidative stress response. In particular, miR-494 and miR-542-3p modulated the expression of cadherin-17 with concomitant upregulation of matrix metalloproteinases. Together, these findings demonstrate that TD-exosomes may target non-transformed cells in pre-metastatic tissues, leading to modulation of gene expression in these cells specifically through transfer of microRNA and priming distant tissues for tumor cell hosting [118].

Exosomal transfer of microRNAs could also induce permanent changes in recipient cell phenotypes via transfer of microRNAs that are known to regulate genes involved in epigenetic reprogramming (*i.e.*, miR-101 regulation of the histone methyltransferase EZH2). For example, it has been demonstrated that microvesicles derived from one cell type can deliver mRNAs that could mediate gene expression and alter cell fate in a secondary recipient cell type (reviewed in [119]). While we are unaware of any studies demonstrating changes in epigenetic programming or cell fate through the exchange of exosomal microRNAs, it is certainly a highly plausible occurrence. In conclusion, it is clear from these studies, summarized in Table 2, that transfer of microRNAs via exosomes is indeed a mechanism of intercellular communication that can initiate and promote tumor progression via transfer of genetic information at local and distant cells and tissues.

## 4. Exosomes and Breast Cancer

### 4.1. Exosomes in Normal Mammary Epithelium

The human mammary gland is comprised of two main compartments: the branching epithelial ductal-lobular system and the supporting stroma. The epithelial component is comprised of two epithelial cell types: the luminal cells, whose function is to maintain the apical-basal polarity within the lumen and to produce and secrete milk into the ducts, and the myoepithelial cells, whose function is to maintain the organization of the mammary gland and to contract and eject the milk in response to hormonal signals. The mammary gland is one of the few organs to undergo significant developmental changes after birth, including growth at the onset of puberty and pregnancy, lactation and regression upon cessation of lactation [120]. Breast milk, which is a complex and nutrient rich liquid that contains proteins, lipids, carbohydrates and trace elements, is known to be the optimal nutrition and an important source of immunoprotective components for infants during their first months of life [121]. Exosomes have been identified in both colostrum and mature human breast milk that have the capacity to potentially influence the immune response in infants [5]. Specifically, breast milk exosomes were found to inhibit anti-CD3-induced IL-2 and interferon (IFN)-γ production and could increase the number of T-regulatory cells from peripheral blood mononuclear cells when incubated with milk vesicle preparations [3]. Furthermore, breast milk exosomes are also known to contain microRNAs [122]. Over 600 unique microRNAs, originating from ~450 microRNA precursors, have been identified in human breast milk exosomes using deep sequencing technology, and greater than 65% of the known immune-related microRNAs were enriched in these exosomes [122]. These results suggest that microRNAs can be transferred from the mother’s milk to the infant via the digestive tract, where they could play an important role in the development of the infant’s immune system, although further work must be done to confirm these speculations. At this time, however, it is unknown if exosomes are secreted in breast luminal epithelial cells during the growth phase of puberty or pregnancy, during regression or during the mammary resting state. It is also unknown what role the myoepithelial cells play in exosome secretion or in crosstalk between the two epithelial lineages and the breast stroma in the normal mammary gland. Furthermore, the aberrant secretion of exosomes and their specific contribution to breast cancer development and early progression are also unknown at this time.’

### 4.2. Exosomes in Breast Cancer Development and Progression

Breast cancer is one of the most frequently observed cancers in industrialized countries and is the second leading cause of cancer death among women in the US. Every year, ~200,000 new cases of invasive breast cancer will be diagnosed, and around 40,000 women are expected to die from breast cancer [123]. The general view of the tumorigenic process involves cells that have acquired critical genetic and epigenetic abnormalities that inhibit their responsiveness to normal growth and regulatory signals. In the mammary gland, the majority of these critical genetic changes occurs in the luminal epithelium at the transition from normal, to hyperplastic, to pre-invasive lesions and contains greater measures of genetic and morphological abnormalities, including aneuploidy [124–126], oncogene amplification [127,128] or allele imbalance [129,130]. It is thought that fewer abnormalities exist in precursor lesions and more are acquired as the cancer progresses. In addition, the loss of the normal myoepithelium cell layer and apical-basal polarity are early signs of tumorigenesis. It has also been demonstrated that the microenvironment also undergoes changes and has a dramatic influence on the tumor, even at the pre-invasive stage [131]. It is suggested that a reciprocal relationship between breast cancer cells and their surrounding microenvironment predominantly influences the energetics and growth of the cancer. However, the role of exosomes in mediating this reciprocal relationship is only beginning to emerge. Early studies in breast cancer exosomes demonstrated that exosomes might play a role in the control of tumor growth. For example, mice pretreated with exosomes derived from murine mammary carcinomas had increased rates of tumor growth, due to inhibition of natural killer cells [132]. In co-culture experiments, tumor-associated macrophages were shown to transfer miR-223, a microRNA specific for IL-4 activated macrophages, into breast cancer cells, where it promoted invasion through activation of the Mef2c-β-catenin pathway [114]. Uptake of the epidermal growth factor receptor (EGFR) ligand, amphiregulin, carried by exosomes to breast cancer cells, increased their invasiveness compared to exosomes carrying other EGFR ligands, suggesting a role for exosomes in the cancer “field effect” and metastatic niche priming [133]. Another study examined the effects of exosomes derived from a triple-negative breast cancer (TNBC) cell line (Hs578T) *versus* its more invasive variant (Hs578T(i)_8_) on three recipient TNBC cell lines. It was shown that exosomes from the more invasive variant increase proliferation, migration, and invasion and stimulate significantly more endothelial tubule formation in all recipient cell lines [134]. The intercellular communication between fibroblasts and breast cancer cells was recently examined, which showed that fibroblast-secreted exosomes could promote breast cancer cell protrusive and motile activity through Wnt-planar cell polarity (PCP) signaling and that co-injection of fibroblasts with breast cancer cells in an orthotopic mouse model could dramatically increase metastasis that was dependent on the PCP pathway [135]. A recent study utilized GFP-tagged CD63 expressing breast cancer cells to examine the fate of cancer-cell derived exosomes in a nude mouse model of breast cancer. They demonstrated that breast cancer cells could transfer their exosomes to other cancer cells and normal lung tissue *in vitro* and into the tumor microenvironment and the circulation of mice with breast cancer metastases *in vivo* [136].

While very few studies thus far have profiled or examined the microRNAs present in breast cancer exosomes, a previously mentioned study has identified a specific set microRNAs that are secreted in exosomes or retained in cells that differ between non-malignant and malignant breast cancer cells [49]. Further studies demonstrated that the selectively exported microRNAs from breast cancer cells are packaged in exosomes that differ from conventional exosomes [117]. However, the mechanism of this selective microRNA transport and altered exosome formation in malignant breast cancer cells is unknown at this time.

In summary, these studies suggest that breast cancer derived-exosomes may contribute significantly to breast tumor growth and development, promotion of angiogenesis, invasion and formation of a pre-metastatic niche to promote tumor growth and metastasis. Moreover, further elucidation of the mechanisms of how exosomes and their residual components mediate the intercellular communication in the breast tumor microenvironment and how this process unfolds during early malignant transformation and cancer progression are of great research interest.

### 4.3. Potential for Diagnostic, Prognostic and Therapeutic Interference

Much of the excitement surrounding TD-exosome research is due to its high clinical relevance. In particular, because breast cancer exosomes can be easily isolated through minimally invasive procedures, such as from the blood or ductal lavage of breast cancer patients, they have great potential in breast cancer diagnosis. Because exosomal microRNA profiles of circulating tumor exosomes tend to be unique from those in normal controls, breast cancer-specific exosomal-microRNA signatures may also be developed to predict tumor development. It is accepted that breast cancer is a highly heterogeneous disease with phenotypically diverse tumors, which have been categorized by their gene expression profiles as the intrinsic molecular subtypes of breast cancer. These are generally defined as: basal-like breast cancer, which generally corresponds to estrogen receptor (ER) negative, progesterone receptor (PR) and HER-2 negative (*i.e.*, triple negative), luminal A (ER positive, low grade), luminal B (ER positive, high grade) and HER-2 (or ErbB2) positive [137]. These subtypes have distinctly different gene and microRNA expression profiles [138,139]. Therefore, their secreted exosomes may also have distinctly different RNA profiles that could correspond to the molecular subtype of their host tumors. Exosomes may also be important players in chemotherapy and chemoresistance. For example, release of exosomes from HER2-overexpressing breast cancer cell lines (BT474 and SKBR3) or from HER2-positive breast cancer patient serum could bind to Trastuzumab (a monoclonal antibody therapy that interferes with the HER2 receptor) and lead to an inhibition of the anti-proliferative effects of Trastuzumab on SKBR3 cells. These results suggest that HER2-positive exosomes may interfere with anticancer therapy and may promote HER-2 driven tumor aggressiveness [140]. These findings have led to the development of a novel therapeutic strategy for exosome removal as an adjuvant to chemotherapy. In particular, Aethlon Medical, Inc. (San Diego, CA, USA) has introduced the HER2osome^TM^ as a new therapeutic strategy to maximize the effects of anti-HER2 therapies in combating breast cancer [141]. Furthermore, exosomes may also function to shuttle chemotherapies, such as cisplatin, out of the tumor cell, thus reducing their effectiveness [142]. For example, acquired resistance to cisplatin is associated with abnormalities of protein trafficking and secretion. Cisplatin-resistant ovarian cancer cells release more protein via exosomes, including greater levels of the lysosome-associated protein 1 (LAMP1), the putative cisplatin export transporter, MRP2, and the copper export proteins, ATP7A and ATP7B, than those released by cisplatin-sensitive cells [142]. This implicates the exosome secretion pathway in the resistance of breast cancer to cancer therapy. Lastly, because exosomes are naturally produced, cell-derived nucleic acid carriers, they also hold the potential to function as biological therapeutic delivery systems. For example, exosomes engineered to express the transmembrane domain of the PDGF receptor fused to the GE11 peptide were shown to successfully deliver the let-7a microRNA to EGFR-expressing breast cancer cells *in vitro* and EGFR-expressing xenograft breast cancer tissue *in vivo*, thus demonstrating the potential of exosomes to be used therapeutically to specifically target cancer cells with nucleic acid drug targets [143].

## 5. Future Directions

Current knowledge of TD-exosomes suggests that they can play an important role in the development and progression of cancer through modulation of intercellular communication within the tumor microenvironment by the transfer of protein, lipid and RNA cargo. A further exploration of exosome secretion in the normal physiological state and during cancer development and progression, as well as the specific content exosomes transport under these conditions, will increase our understanding of their role in intercellular communication and tumorigenesis. Identification and modification of cancer cell-derived exosome contents may allow for the development of novel diagnostic, preventive and therapeutic approaches, with potentially minimally invasive procedures. Utilization of exosomes for therapeutic delivery may also prove to be the answer for the field of RNA therapeutics, whose main roadblock has been the development of an effective RNA delivery system. Furthermore, the concept of exploiting these extracellular vesicles or the creation of synthetic exosomes, called “exosome mimetics” for drug delivery may also allow for specific targeting of cancer cells by developing exosomes that harbor cell-specific targeting factors [144]. Although this field is in its infancy, it is easy to imagine all the future possibilities these natural nanoparticles hold.

## Figures and Tables

**Table 1:** Summary of clinical microRNA profiling studies of circulating tumor exosomes/microvesicles.

Cancer type	Clinical samples	Exosome isolation method	Major findings	Potential diagnostic microRNAs	Reference
Ovarian cancer	Sera from patients with serous papillary adenocarcinoma (*n* = 50); sera from age-matched controls with benign ovarian adenoma (*n* = 10); primary ovarian adenocarcinoma tumor cell cultures and matched patient sera (*n* = 6).	Magnetic activated cell sorting using beads coupled with anti-EpCAM.	Exosomal microRNA profiles were similar in ovarian cancer patient samples and distinctly different from benign disease samples. microRNAs were elevated in exosomes *versus* tumor cells (31 out of 467).	miR-21, miR-141, miR-200a, miR-200c, miR-203, miR-205 and miR-214	[9]
Lung adenocarcinoma	Plasma from patients with lung adenocarcinoma (*n* = 27); plasma from controls (*n* = 9); matched plasma and lung tumor tissue (*n* = 4).	Size exclusion chromatography and magnetically activated cell sorting using beads coupled with anti-EpCAM.	No significant differences in exosome microRNA levels between microRNAs derived from circulating exosomes or from microRNAs from the primary tumor were observed.	miR-17-3p, miR-21, miR-106a, miR-146, miR-155, miR-191, miR-192, miR-203, miR-205, miR-210, miR-212, miR-214	[48]
Prostate cancer	Plasma from prostate cancer patients (*n* = 78); plasma from normal controls (*n* = 28); urine samples (*n* = 135); serum from patients with recurrent metastatic prostate cancer (*n* = 47) or non-recurrent disease (*n* = 72).	Filtration of plasma through a 1.2 μm filter, concentrated with a 150 kDa molecular weight cut-off.	The levels of 12 microRNAs were different between plasma exosomes of prostate cancer patients compared to control. Eleven microRNAs were present in significantly greater amounts in patients with metastases *versus* without. The association of exosomal miR-141 and miR-375 with metastases was confirmed in a second patient population.	miR-107, miR-130b, miR-141, miR-181a-2*, miR-2110, miR- 301a, miR-326, miR-331-3p, miR-432, miR-438, miR-574-3p, miR-625*	[110]
Esophageal squamous cell carcinoma	Serum from ESCC patients (*n* = 101); Serum from healthy controls (*n* = 46).	Sequential centrifugation, 0.22 μm filtration and ultracentrifugation.	miR-1246 was markedly elevated in serum and exosomes from ESCC patients and was a strong independent risk factor for poor survival. miR-1246 expression was not increased in ESCC tissue samples.	miR-1246	[111]

Abbreviations: EpCAM = epithelial cell adhesion molecule; ESCC = esophageal squamous cell carcinoma.

**Table 2:** Summary of *in vitro* studies of microRNAs derived from cancer cell exosomes.

Cell Line model	Major findings	Predominant microRNAs	Target genes or pathways	Reference
Human and mouse mast cells	Identified small RNAs, including 121 microRNAs and 1,300 specific mRNAs. Detected mouse exosomal RNA and new mouse proteins in human mast cells after treatment with mouse mast cell exosomes. Coined the term “exosomal shuttle RNA (esRNA)”.	let-7, miR-1, miR-15, miR-16, miR-181, miR-375.	None tested	[45]
Metastatic gastric cell line	Profiled microRNA expression by microarray in exosomes isolated from gastric cancer cells. let-7 microRNA family was enriched in exosomes.	let-7 family	None tested	[106]
Co-culture of IL-4-activated macrophages and breast cancer cells	miRNAs can be transferred from macrophages to breast cancer cells. miR-223 released by macrophages was found in MCF7 and MDA-MB-231 cells and promoted invasion.	miR-223	Mef2c-β-catenin pathway	[114]
Mouse dendritic cells	Exosomal microRNA from dendritic cells can be transferred to a recipient dendritic cell and repress microRNA target mRNAs in the acceptor cell.	miR-148a, miR-451	Luciferase reporter containing tandem microRNA target sequences	[108]
Leukemia cells and endothelial cells	Leukemia cells released microRNAs from the miR-17-92 cluster and were taken up by human umbilical vein endothelial cells (HUVECs) and repressed a target mRNA. Did not affect the growth of HUVEC cells, but did enhance cell migration and tube formation.	miR-92a	Integrin α5	[113]
Hepatocellular carcinoma cells	Transmission of exosome microRNAs from hepatocellular carcinoma cells could contribute to the initiation and progression of hepatocellular carcinoma by targeting a tumor suppressor frequently lost in hepatocarcinogenesis.	miR-584, miR-517c, miR-378, miR-520f, miR-142-5p, miR-451, miR-518d, miR-215, miR-376a*, miR-133b, miR-367	Transforming growth factor β activated kinase-1 (TAK1) pathway	[109]
Renal cancer stem cells	Microvesicles were secreted from human renal cell carcinoma that could trigger angiogenesis and premetastatic niche formation in a severe combined immunodeficient (SCID) mouse model.	miR-92, miR-141, miR-29a, miR-650, miR-151, miR-19b, miR-29c	Increase in VEGFR1 and MMP-9 expression	[87]
Breast cancer cells	Selective release of certain microRNA populations was demonstrated in malignant breast cancer cells that are retained in non-malignant mammary epithelial cells.	miR-451, miR-1246	None tested	[49]
Metastatic rat adenocarcinoma cells	Exosomes were preferentially taken up by lymph node stroma cells and lung fibroblasts. The transferred microRNAs affected mRNA translation of many genes.	miR-494, miR-542-3p	Cadherin-17 and many proteases, adhesion molecules, chemokine ligands, cell cycle- and angiogenesis-promoting and oxidative stress response genes.	[118]
Lung cancer cell lines	miR-21 and miR-29a were secreted in exosomes and could bind to murine TLR7 and human TLR8 and trigger a Toll-like receptor (TLR)-mediated prometastatic inflammatory response that could lead to tumor growth and metastasis.	miR-21 and miR-29a	Toll-like receptor (TLR) 8 and 9	[116]
Melanoma and normal melanocyte cells	The first in-depth screening to examine the entire exosome transcriptome, miRNome and proteome. Thousands of mRNAs and 15 microRNAs that are associated with melanoma progression and metastasis were identified.	let-7 family, miR-138, miR-125b, miR-130a, miR-34a, miR-196a, miR-199b-3p, miR-25, miR-27a, miR-200b, miR-23b, miR-146a, miR-613, miR-205, miR-149	None tested	[51]

## References

[1] Thery C., Zitvogel L., Amigorena S. (2002). Exosomes: Composition, biogenesis and function. Nat. Rev. Immunol.

[2] Wickman G., Julian L., Olson M.F. (2012). How apoptotic cells aid in the removal of their own cold dead bodies. Cell Death Differ.

[3] Pan B.T., Teng K., Wu C., Adam M., Johnstone R.M. (1985). Electron microscopic evidence for externalization of the transferrin receptor in vesicular form in sheep reticulocytes. J. Cell Biol.

[4] Harding C., Heuser J., Stahl P. (1983). Receptor-mediated endocytosis of transferrin and recycling of the transferrin receptor in rat reticulocytes. J. Cell Biol.

[5] Admyre C., Johansson S.M., Qazi K.R., Filen J.J., Lahesmaa R., Norman M., Neve E.P., Scheynius A., Gabrielsson S. (2007). Exosomes with immune modulatory features are present in human breast milk. J. Immunol.

[6] Masyuk A.I., Masyuk T.V., Larusso N.F. (2013). Exosomes in the pathogenesis, diagnostics and therapeutics of liver diseases. J. Hepatol..

[7] Vella L.J., Sharples R.A., Nisbet R.M., Cappai R., Hill A.F. (2008). The role of exosomes in the processing of proteins associated with neurodegenerative diseases. Eur. Biophys. J.

[8] Keller S., Rupp C., Stoeck A., Runz S., Fogel M., Lugert S., Hager H.D., Abdel-Bakky M.S., Gutwein P., Altevogt P. (2007). CD24 is a marker of exosomes secreted into urine and amniotic fluid. Kidney Int.

[9] Taylor D.D., Gercel-Taylor C. (2008). MicroRNA signatures of tumor-derived exosomes as diagnostic biomarkers of ovarian cancer. Gynecol. Oncol.

[10] Gallo A., Tandon M., Alevizos I., Illei G.G. (2012). The majority of microRNAs detectable in serum and saliva is concentrated in exosomes. PLoS One.

[11] Saman S., Kim W., Raya M., Visnick Y., Miro S., Saman S., Jackson B., McKee A.C., Alvarez V.E., Lee N.C. (2012). Exosome-associated tau is secreted in tauopathy models and is selectively phosphorylated in cerebrospinal fluid in early Alzheimer disease. J. Biol. Chem.

[12] Qiu S., Duan X., Geng X., Xie J., Gao H. (2012). Antigen-specific activities of CD8+ T cells in the nasal mucosa of patients with nasal allergy. Asian Pac. J. Allergy Immunol. Launched Allergy Immunol. Soc. Thail.

[13] Record M., Zhang H.-G. (2013). Exosomal Lipids in Cell–Cell Communication. Emerging Concepts of Tumor Exosome–Mediated Cell-Cell Communication.

[14] Bobrie A., Colombo M., Raposo G., Thery C. (2011). Exosome secretion: Molecular mechanisms and roles in immune responses. Traffic.

[15] Hanson P.I., Cashikar A. (2012). Multivesicular body morphogenesis. Annu. Rev. Cell Dev. Biol.

[16] Schmidt O., Teis D. (2012). The ESCRT machinery. Curr. Biol.

[17] Raymond C.K., Howald-Stevenson I., Vater C.A., Stevens T.H. (1992). Morphological classification of the yeast vacuolar protein sorting mutants: Evidence for a prevacuolar compartment in class E vps mutants. Mol. Biol. Cell.

[18] Raiborg C., Stenmark H. (2009). The ESCRT machinery in endosomal sorting of ubiquitylated membrane proteins. Nature.

[19] Katzmann D.J., Babst M., Emr S.D. (2001). Ubiquitin-dependent sorting into the multivesicular body pathway requires the function of a conserved endosomal protein sorting complex, ESCRT-I. Cell.

[20] Wollert T., Wunder C., Lippincott-Schwartz J., Hurley J.H. (2009). Membrane scission by the ESCRT-III complex. Nature.

[21] Van Blitterswijk W.J., van der Luit A.H., Veldman R.J., Verheij M., Borst J. (2003). Ceramide: Second messenger or modulator of membrane structure and dynamics?. Biochem. J.

[22] Subra C., Laulagnier K., Perret B., Record M. (2007). Exosome lipidomics unravels lipid sorting at the level of multivesicular bodies. Biochimie.

[23] Trajkovic K., Hsu C., Chiantia S., Rajendran L., Wenzel D., Wieland F., Schwille P., Brugger B., Simons M. (2008). Ceramide triggers budding of exosome vesicles into multivesicular endosomes. Science.

[24] Van Niel G., Charrin S., Simoes S., Romao M., Rochin L., Saftig P., Marks M.S., Rubinstein E., Raposo G. (2011). The tetraspanin CD63 regulates ESCRT-independent and -dependent endosomal sorting during melanogenesis. Dev. Cell.

[25] Rodriguez-Boulan E., Kreitzer G., Musch A. (2005). Organization of vesicular trafficking in epithelia. Nat. Rev. Mol. Cell Biol.

[26] Hutagalung A.H., Novick P.J. (2011). Role of Rab GTPases in membrane traffic and cell physiology. Physiol. Rev.

[27] Savina A., Vidal M., Colombo M.I. (2002). The exosome pathway in K562 cells is regulated by Rab11. J. Cell Sci.

[28] Ostrowski M., Carmo N.B., Krumeich S., Fanget I., Raposo G., Savina A., Moita C.F., Schauer K., Hume A.N., Freitas R.P. (2010). Rab27a and Rab27b control different steps of the exosome secretion pathway. Nat. Cell Biol.

[29] Yu X., Harris S.L., Levine A.J. (2006). The regulation of exosome secretion: A novel function of the p53 protein. Cancer Res.

[30] King H.W., Michael M.Z., Gleadle J.M. (2012). Hypoxic enhancement of exosome release by breast cancer cells. BMC Cancer.

[31] Thompson C.A., Purushothaman A., Ramani V.C., Vlodavsky I., Sanderson R.D. (2013). Heparanase Regulates Secretion, Composition, and Function of Tumor Cell-derived Exosomes. J. Biol. Chem.

[32] Koumangoye R.B., Sakwe A.M., Goodwin J.S., Patel T., Ochieng J. (2011). Detachment of breast tumor cells induces rapid secretion of exosomes which subsequently mediate cellular adhesion and spreading. PLoS One.

[33] Llorente A., van Deurs B., Sandvig K. (2007). Cholesterol regulates prostasome release from secretory lysosomes in PC-3 human prostate cancer cells. Eur. J. Cell Biol.

[34] Faure J., Lachenal G., Court M., Hirrlinger J., Chatellard-Causse C., Blot B., Grange J., Schoehn G., Goldberg Y., Boyer V. (2006). Exosomes are released by cultured cortical neurones. Mol. Cell. Neurosci.

[35] Parolini I., Federici C., Raggi C., Lugini L., Palleschi S., de Milito A., Coscia C., Iessi E., Logozzi M., Molinari A. (2009). Microenvironmental pH is a key factor for exosome traffic in tumor cells. J. Biol. Chem.

[36] Mittelbrunn M., Gutierrez-Vazquez C., Villarroya-Beltri C., Gonzalez S., Sanchez-Cabo F., Gonzalez M.A., Bernad A., Sanchez-Madrid F. (2011). Unidirectional transfer of microRNA-loaded exosomes from T cells to antigen-presenting cells. Nat. Commun.

[37] Keller S., Konig A.K., Marme F., Runz S., Wolterink S., Koensgen D., Mustea A., Sehouli J., Altevogt P. (2009). Systemic presence and tumor-growth promoting effect of ovarian carcinoma released exosomes. Cancer Lett.

[38] Barres C., Blanc L., Bette-Bobillo P., Andre S., Mamoun R., Gabius H.J., Vidal M. (2010). Galectin-5 is bound onto the surface of rat reticulocyte exosomes and modulates vesicle uptake by macrophages. Blood.

[39] Mathivanan S., Fahner C.J., Reid G.E., Simpson R.J. (2012). ExoCarta 2012: Database of exosomal proteins, RNA and lipids. Nucleic Acids Res.

[40] Mathivanan S., Simpson R.J. (2009). ExoCarta: A compendium of exosomal proteins and RNA. Proteomics.

[41] Subra C., Grand D., Laulagnier K., Stella A., Lambeau G., Paillasse M., de Medina P., Monsarrat B., Perret B., Silvente-Poirot S. (2010). Exosomes account for vesicle-mediated transcellular transport of activatable phospholipases and prostaglandins. J. Lipid Res.

[42] Zakharova L., Svetlova M., Fomina A.F. (2007). T cell exosomes induce cholesterol accumulation in human monocytes via phosphatidylserine receptor. J. Cell. Physiol.

[43] Xiang X., Poliakov A., Liu C., Liu Y., Deng Z.B., Wang J., Cheng Z., Shah S.V., Wang G.J., Zhang L. (2009). Induction of myeloid-derived suppressor cells by tumor exosomes. Int. J. Cancer.

[44] Cao W., Ma Z., Rasenick M.M., Yeh S., Yu J. (2012). N-3 poly-unsaturated fatty acids shift estrogen signaling to inhibit human breast cancer cell growth. PLoS One.

[45] Valadi H., Ekstrom K., Bossios A., Sjostrand M., Lee J.J., Lotvall J.O. (2007). Exosome-mediated transfer of mRNAs and microRNAs is a novel mechanism of genetic exchange between cells. Nat. Cell Biol.

[46] Hessvik N.P., Phuyal S., Brech A., Sandvig K., Llorente A. (2012). Profiling of microRNAs in exosomes released from PC-3 prostate cancer cells. Biochim. Biophys. Acta.

[47] Bellingham S.A., Coleman B.M., Hill A.F. (2012). Small RNA deep sequencing reveals a distinct miRNA signature released in exosomes from prion-infected neuronal cells. Nucleic Acids Res.

[48] Rabinowits G., Gercel-Taylor C., Day J.M., Taylor D.D., Kloecker G.H. (2009). Exosomal microRNA: A diagnostic marker for lung cancer. Clin. Lung Cancer.

[49] Pigati L., Yaddanapudi S.C., Iyengar R., Kim D.J., Hearn S.A., Danforth D., Hastings M.L., Duelli D.M. (2010). Selective release of microRNA species from normal and malignant mammary epithelial cells. PLoS One.

[50] Jaiswal R., Luk F., Gong J., Mathys J.M., Grau G.E., Bebawy M. (2012). Microparticle conferred microRNA profiles—Implications in the transfer and dominance of cancer traits. Mol. Cancer.

[51] Xiao D., Ohlendorf J., Chen Y., Taylor D.D., Rai S.N., Waigel S., Zacharias W., Hao H., McMasters K.M. (2012). Identifying mRNA, microRNA and protein profiles of melanoma exosomes. PLoS One.

[52] Simpson R.J., Jensen S.S., Lim J.W. (2008). Proteomic profiling of exosomes: Current perspectives. Proteomics.

[53] de Gassart A., Geminard C., Fevrier B., Raposo G., Vidal M. (2003). Lipid raft-associated protein sorting in exosomes. Blood.

[54] Thery C., Amigorena S., Raposo G., Clayton A. (2006). Isolation and characterization of exosomes from cell culture supernatants and biological fluids. Curr. Protoc. Cell Biol..

[55] Cantin R., Diou J., Belanger D., Tremblay A.M., Gilbert C. (2008). Discrimination between exosomes and HIV-1: Purification of both vesicles from cell-free supernatants. J. Immunol. Methods.

[56] Lamparski H.G., Metha-Damani A., Yao J.Y., Patel S., Hsu D.H., Ruegg C., Le Pecq J.B. (2002). Production and characterization of clinical grade exosomes derived from dendritic cells. J. Immunol. Methods.

[57] System Biosciences http://www.systembio.com/exoquick.

[58] Taylor D.D., Atay S., Metzinger D.S., Gercel-Taylor C. (2010). Characterization of humoral responses of ovarian cancer patients: Antibody subclasses and antigenic components. Gynecol. Oncol.

[59] Koga K., Matsumoto K., Akiyoshi T., Kubo M., Yamanaka N., Tasaki A., Nakashima H., Nakamura M., Kuroki S., Tanaka M., Katano M. (2005). Purification, characterization and biological significance of tumor-derived exosomes. Anticancer Res.

[60] Tauro B.J., Greening D.W., Mathias R.A., Ji H., Mathivanan S., Scott A.M., Simpson R.J. (2012). Comparison of ultracentrifugation, density gradient separation, and immunoaffinity capture methods for isolating human colon cancer cell line LIM1863-derived exosomes. Methods.

[61] Taylor D.D., Zacharias W., Gercel-Taylor C. (2011). Exosome isolation for proteomic analyses and RNA profiling. Methods Mol. Biol.

[62] Simona F., Laura S., Simona T., Riccardo A. (2013). Contribution of proteomics to understanding the role of tumor-derived exosomes in cancer progression: State of the art and new perspectives. Proteomics.

[63] Eldh M., Lotvall J., Malmhall C., Ekstrom K. (2012). Importance of RNA isolation methods for analysis of exosomal RNA: Evaluation of different methods. Mol. Immunol.

[64] Lasser C., Eldh M., Lotvall J. (2012). Isolation and characterization of RNA-containing exosomes. J. Vis. Exp..

[65] Lasser C. (2013). Identification and analysis of circulating exosomal microRNA in human body fluids. Methods Mol. Biol.

[66] Huang X., Yuan T., Tschannen M., Sun Z., Jacob H., Du M., Liang M., Dittmar R.L., Liu Y., Liang M. (2013). Characterization of human plasma-derived exosomal RNAs by deep sequencing. BMC Genomics.

[67] Nanosight http://www.nanosight.com.

[68] Zheng Y., Campbell E.C., Lucocq J., Riches A., Powis S.J. (2012). Monitoring the Rab27 associated exosome pathway using nanoparticle tracking analysis. Exp. Cell Res..

[69] Hanahan D., Coussens L.M. (2012). Accessories to the crime: Functions of cells recruited to the tumor microenvironment. Cancer Cell.

[70] Tian T., Wang Y., Wang H., Zhu Z., Xiao Z. (2010). Visualizing of the cellular uptake and intracellular trafficking of exosomes by live-cell microscopy. J. Cell. Biochem.

[71] Ristorcelli E., Beraud E., Verrando P., Villard C., Lafitte D., Sbarra V., Lombardo D., Verine A. (2008). Human tumor nanoparticles induce apoptosis of pancreatic cancer cells. FASEB J.

[72] Yang Y., Xiu F., Cai Z., Wang J., Wang Q., Fu Y., Cao X. (2007). Increased induction of antitumor response by exosomes derived from interleukin-2 gene-modified tumor cells. J. Cancer Res. Clin. Oncol.

[73] Dai S., Wei D., Wu Z., Zhou X., Wei X., Huang H., Li G. (2008). Phase I clinical trial of autologous ascites-derived exosomes combined with GM-CSF for colorectal cancer. Mol. Ther.

[74] Zhang Y., Wu X.H., Luo C.L., Zhang J.M., He B.C., Chen G. (2010). Interleukin-12-anchored exosomes increase cytotoxicity of T lymphocytes by reversing the JAK/STAT pathway impaired by tumor-derived exosomes. Int. J. Mol. Med.

[75] Zhang Y., Luo C.L., He B.C., Zhang J.M., Cheng G., Wu X.H. (2010). Exosomes derived from IL-12-anchored renal cancer cells increase induction of specific antitumor response *in vitro*: A novel vaccine for renal cell carcinoma. Int. J. Oncol.

[76] Chen T., Guo J., Yang M., Zhu X., Cao X. (2011). Chemokine-containing exosomes are released from heat-stressed tumor cells via lipid raft-dependent pathway and act as efficient tumor vaccine. J. Immunol.

[77] Peng P., Yan Y., Keng S. (2011). Exosomes in the ascites of ovarian cancer patients: Origin and effects on anti-tumor immunity. Oncol. Rep.

[78] Hakulinen J., Sankkila L., Sugiyama N., Lehti K., Keski-Oja J. (2008). Secretion of active membrane type 1 matrix metalloproteinase (MMP-14) into extracellular space in microvesicular exosomes. J. Cell. Biochem.

[79] McCready J., Sims J.D., Chan D., Jay D.G. (2010). Secretion of extracellular hsp90alpha via exosomes increases cancer cell motility: A role for plasminogen activation. BMC Cancer.

[80] Janowska-Wieczorek A., Wysoczynski M., Kijowski J., Marquez-Curtis L., Machalinski B., Ratajczak J., Ratajczak M.Z. (2005). Microvesicles derived from activated platelets induce metastasis and angiogenesis in lung cancer. Int. J. Cancer.

[81] Cho J.A., Park H., Lim E.H., Kim K.H., Choi J.S., Lee J.H., Shin J.W., Lee K.W. (2011). Exosomes from ovarian cancer cells induce adipose tissue-derived mesenchymal stem cells to acquire the physical and functional characteristics of tumor-supporting myofibroblasts. Gynecol. Oncol.

[82] Bobrie A., Krumeich S., Reyal F., Recchi C., Moita L.F., Seabra M.C., Ostrowski M., Thery C. (2012). Rab27a supports exosome-dependent and -independent mechanisms that modify the tumor microenvironment and can promote tumor progression. Cancer Res.

[83] Park J.E., Tan H.S., Datta A., Lai R.C., Zhang H., Meng W., Lim S.K., Sze S.K. (2010). Hypoxic tumor cell modulates its microenvironment to enhance angiogenic and metastatic potential by secretion of proteins and exosomes. Mol. Cell. Proteomics.

[84] Kucharzewska P., Christianson H.C., Welch J.E., Svensson K.J., Fredlund E., Ringner M., Morgelin M., Bourseau-Guilmain E., Bengzon J., Belting M. (2013). Exosomes reflect the hypoxic status of glioma cells and mediate hypoxia-dependent activation of vascular cells during tumor development. Proc. Natl. Acad. Sci. USA.

[85] Hong B.S., Cho J.H., Kim H., Choi E.J., Rho S., Kim J., Kim J.H., Choi D.S., Kim Y.K., Hwang D. (2009). Colorectal cancer cell-derived microvesicles are enriched in cell cycle-related mRNAs that promote proliferation of endothelial cells. BMC Genomics.

[86] Ji H., Greening D.W., Barnes T.W., Lim J.W., Tauro B.J., Rai A., Xu R., Adda C., Mathivanan S., Zhao W. (2013). Proteome profiling of exosomes derived from human primary and metastatic colorectal cells reveal differential expression of key metastatic factors and signal transduction components. Proteomics.

[87] Grange C., Tapparo M., Collino F., Vitillo L., Damasco C., Deregibus M.C., Tetta C., Bussolati B., Camussi G. (2011). Microvesicles released from human renal cancer stem cells stimulate angiogenesis and formation of lung premetastatic niche. Cancer Res.

[88] Zhu W., Huang L., Li Y., Zhang X., Gu J., Yan Y., Xu X., Wang M., Qian H., Xu W. (2012). Exosomes derived from human bone marrow mesenchymal stem cells promote tumor growth *in vivo*. Cancer Lett.

[89] Mineo M., Garfield S.H., Taverna S., Flugy A., De Leo G., Alessandro R., Kohn E.C. (2012). Exosomes released by K562 chronic myeloid leukemia cells promote angiogenesis in a Src-dependent fashion. Angiogenesis.

[90] Peinado H., Aleckovic M., Lavotshkin S., Matei I., Costa-Silva B., Moreno-Bueno G., Hergueta-Redondo M., Williams C., Garcia-Santos G., Ghajar C. (2012). Melanoma exosomes educate bone marrow progenitor cells toward a pro-metastatic phenotype through MET. Nat. Med.

[91] Baer C., Squadrito M.L., Iruela-Arispe M.L., De Palma M. (2013). Reciprocal interactions between endothelial cells and macrophages in angiogenic vascular niches. Exp. Cell Res..

[92] Cai X., Hagedorn C.H., Cullen B.R. (2004). Human microRNAs are processed from capped, polyadenylated transcripts that can also function as mRNAs. RNA.

[93] Lee Y., Jeon K., Lee J.T., Kim S., Kim V.N. (2002). MicroRNA maturation: Stepwise processing and subcellular localization. EMBO J.

[94] Lee Y., Kim M., Han J., Yeom K.H., Lee S., Baek S.H., Kim V.N. (2004). MicroRNA genes are transcribed by RNA polymerase II. EMBO J.

[95] Zeng Y., Cullen B.R. (2004). Structural requirements for pre-microRNA binding and nuclear export by Exportin 5. Nucleic Acids Res.

[96] Bartel D.P. (2009). MicroRNAs: Target recognition and regulatory functions. Cell.

[97] Griffiths-Jones S. (2004). The microRNA registry. Nucleic Acids Res.

[98] Lim L.P., Lau N.C., Garrett-Engele P., Grimson A., Schelter J.M., Castle J., Bartel D.P., Linsley P.S., Johnson J.M. (2005). Microarray analysis shows that some microRNAs downregulate large numbers of target mRNAs. Nature.

[99] Taft R.J., Pang K.C., Mercer T.R., Dinger M., Mattick J.S. (2010). Non-coding RNAs: Regulators of disease. J. Pathol.

[100] Melo S.A., Esteller M. (2011). Dysregulation of microRNAs in cancer: Playing with fire. FEBS Lett.

[101] Lu J., Getz G., Miska E.A., Alvarez-Saavedra E., Lamb J., Peck D., Sweet-Cordero A., Ebert B.L., Mak R.H., Ferrando A.A. (2005). microRNA expression profiles classify human cancers. Nature.

[102] Guo Y., Chen Z., Zhang L., Zhou F., Shi S., Feng X., Li B., Meng X., Ma X., Luo M. (2008). Distinctive microRNA profiles relating to patient survival in esophageal squamous cell carcinoma. Cancer Res.

[103] Gibbings D.J., Ciaudo C., Erhardt M., Voinnet O. (2009). Multivesicular bodies associate with components of miRNA effector complexes and modulate miRNA activity. Nat. Cell Biol.

[104] Kosaka N., Iguchi H., Yoshioka Y., Takeshita F., Matsuki Y., Ochiya T. (2010). Secretory mechanisms and intercellular transfer of microRNAs in living cells. J. Biol. Chem.

[105] Yao B., La L.B., Chen Y.C., Chang L.J., Chan E.K. (2012). Defining a new role of GW182 in maintaining miRNA stability. EMBO Rep.

[106] Ohshima K., Inoue K., Fujiwara A., Hatakeyama K., Kanto K., Watanabe Y., Muramatsu K., Fukuda Y., Ogura S., Yamaguchi K. (2010). Let-7 microRNA family is selectively secreted into the extracellular environment via exosomes in a metastatic gastric cancer cell line. PLoS One.

[107] Cifuentes D., Xue H., Taylor D.W., Patnode H., Mishima Y., Cheloufi S., Ma E., Mane S., Hannon G.J., Lawson N.D. (2010). A novel miRNA processing pathway independent of Dicer requires Argonaute2 catalytic activity. Science.

[108] Montecalvo A., Larregina A.T., Shufesky W.J., Stolz D.B., Sullivan M.L., Karlsson J.M., Baty C.J., Gibson G.A., Erdos G., Wang Z. (2012). Mechanism of transfer of functional microRNAs between mouse dendritic cells via exosomes. Blood.

[109] Kogure T., Lin W.L., Yan I.K., Braconi C., Patel T. (2011). Intercellular nanovesicle-mediated microRNA transfer: A mechanism of environmental modulation of hepatocellular cancer cell growth. Hepatology.

[110] Bryant R.J., Pawlowski T., Catto J.W., Marsden G., Vessella R.L., Rhees B., Kuslich C., Visakorpi T., Hamdy F.C. (2012). Changes in circulating microRNA levels associated with prostate cancer. Br. J. Cancer.

[111] Takeshita N., Hoshino I., Mori M., Akutsu Y., Hanari N., Yoneyama Y., Ikeda N., Isozaki Y., Maruyama T., Akanuma N. (2013). Serum microRNA expression profile: miR-1246 as a novel diagnostic and prognostic biomarker for oesophageal squamous cell carcinoma. Br. J. Cancer.

[112] Russo F., di Bella S., Nigita G., Macca V., Lagana A., Giugno R., Pulvirenti A., Ferro A. (2012). miRandola: Extracellular circulating microRNAs database. PLoS One.

[113] Umezu T., Ohyashiki K., Kuroda M., Ohyashiki J.H. (2012). Leukemia cell to endothelial cell communication via exosomal miRNAs. Oncogene.

[114] Yang M., Chen J., Su F., Yu B., Su F., Lin L., Liu Y., Huang J.D., Song E. (2011). Microvesicles secreted by macrophages shuttle invasion-potentiating microRNAs into breast cancer cells. Mol. Cancer.

[115] Zhou R., O’Hara S.P., Chen X.M. (2011). MicroRNA regulation of innate immune responses in epithelial cells. Cell. Mol. Immunol.

[116] Fabbri M., Paone A., Calore F., Galli R., Gaudio E., Santhanam R., Lovat F., Fadda P., Mao C., Nuovo G.J. (2012). MicroRNAs bind to Toll-like receptors to induce prometastatic inflammatory response. Proc. Natl. Acad. Sci. USA.

[117] Palma J., Yaddanapudi S.C., Pigati L., Havens M.A., Jeong S., Weiner G.A., Weimer K.M., Stern B., Hastings M.L., Duelli D.M. (2012). MicroRNAs are exported from malignant cells in customized particles. Nucleic Acids Res.

[118] Rana S., Malinowska K., Zoller M. (2013). Exosomal tumor microRNA modulates premetastatic organ cells. Neoplasia.

[119] Quesenberry P.J., Aliotta J.M. (2010). Cellular phenotype switching and microvesicles. Adv. Drug Deliv. Rev.

[120] Weigelt B., Bissell M.J. (2008). Unraveling the microenvironmental influences on the normal mammary gland and breast cancer. Semin. Cancer Biol.

[121] (2012). Section on Breastfeeding. Breastfeeding and the use of human milk. Pediatrics.

[122] Zhou Q., Li M., Wang X., Li Q., Wang T., Zhu Q., Zhou X., Wang X., Gao X., Li X. (2012). Immune-related microRNAs are abundant in breast milk exosomes. Int. J. Biol. Sci.

[123] Society, A.C. Breast Cancer Facts & Figures 2011–2012.

[124] Locker A., Horrocks C., Gilmour A., Ellis I., Dowle C., Elston C., Blamey R. (1990). Flow cytometric and histological analysis of ductal carcinoma in situ of the breast. Br. J. Surg.

[125] Ottesen G., Christensen I., Larsen J., Christiansen J., Hansen B., Andersen J. (1995). DNA analysis of in situ ductal carcinoma of the breast via flow cytometry. Cytometry.

[126] Leal C., Schmitt F., Bento M., Maia N., Lopes C. (1995). Ductal carcinoma in situ of the breast. Histologic categorization and its relationship to ploidy and immunohistochemical expression of hormone receptors, p53, and c-erbB-2 protein. Cancer.

[127] Maguire H., Hellman M., Greene M., Yeh I. (1992). Expression of c-erbB-2 in in situ and in adjacent invasive ductal adenocarcinomas of the female breast. Pathobiology.

[128] Vos C., Ter Haar N., Peterse J., Cornelisse C., van de Vijver M. (1999). Cyclin D1 gene amplification and overexpression are present in ductal carcinoma in situ of the breast. J. Pathol.

[129] Radford D., Fair K., Phillips N., Ritter J., Steinbrueck T., Holt M., Donis-Keller H. (1995). Allelotyping of ductal carcinoma in situ of the breast: Deletion of loci on 8p, 13q, 16q, 17p and 17q. Cancer Res.

[130] Radford D., Phillips N., Fair K., Ritter J., Holt M., Donis-Keller H. (1995). Allelic loss and the progression of breast cancer. Cancer Res.

[131] Ma X.J., Dahiya S., Richardson E., Erlander M., Sgroi D.C. (2009). Gene expression profiling of the tumor microenvironment during breast cancer progression. Breast Cancer Res.

[132] Liu C., Yu S., Zinn K., Wang J., Zhang L., Jia Y., Kappes J.C., Barnes S., Kimberly R.P., Grizzle W.E. (2006). Murine mammary carcinoma exosomes promote tumor growth by suppression of NK cell function. J. Immunol.

[133] Higginbotham J.N., Demory Beckler M., Gephart J.D., Franklin J.L., Bogatcheva G., Kremers G.J., Piston D.W., Ayers G.D., McConnell R.E., Tyska M.J. (2011). Amphiregulin exosomes increase cancer cell invasion. Curr. Biol.

[134] O’Brien K., Rani S., Corcoran C., Wallace R., Hughes L., Friel A.M., McDonnell S., Crown J., Radomski M.W., O’Driscoll L. (2013). Exosomes from triple-negative breast cancer cells can transfer phenotypic traits representing their cells of origin to secondary cells. Eur. J. Cancer.

[135] Luga V., Zhang L., Viloria-Petit A.M., Ogunjimi A.A., Inanlou M.R., Chiu E., Buchanan M., Hosein A.N., Basik M., Wrana J.L. (2012). Exosomes mediate stromal mobilization of autocrine Wnt-PCP signaling in breast cancer cell migration. Cell.

[136] Suetsugu A., Honma K., Saji S., Moriwaki H., Ochiya T., Hoffman R.M. (2013). Imaging exosome transfer from breast cancer cells to stroma at metastatic sites in orthotopic nude-mouse models. Adv. Drug Deliv. Rev.

[137] Sotiriou C., Pusztai L. (2009). Gene-expression signatures in breast cancer. N. Engl. J. Med.

[138] Blenkiron C., Goldstein L.D., Thorne N.P., Spiteri I., Chin S.F., Dunning M.J., Barbosa-Morais N.L., Teschendorff A.E., Green A.R., Ellis I.O. (2007). MicroRNA expression profiling of human breast cancer identifies new markers of tumor subtype. Genome Biol.

[139] Perou C.M., Sorlie T., Eisen M.B., van de Rijn M., Jeffrey S.S., Rees C.A., Pollack J.R., Ross D.T., Johnsen H., Akslen L.A. (2000). Molecular portraits of human breast tumours. Nature.

[140] Ciravolo V., Huber V., Ghedini G.C., Venturelli E., Bianchi F., Campiglio M., Morelli D., Villa A., Della Mina P., Menard S. (2012). Potential role of HER2-overexpressing exosomes in countering trastuzumab-based therapy. J. Cell. Physiol.

[141] Marleau A.M., Chen C.S., Joyce J.A., Tullis R.H. (2012). Exosome removal as a therapeutic adjuvant in cancer. J. Transl. Med.

[142] Safaei R., Larson B.J., Cheng T.C., Gibson M.A., Otani S., Naerdemann W., Howell S.B. (2005). Abnormal lysosomal trafficking and enhanced exosomal export of cisplatin in drug-resistant human ovarian carcinoma cells. Mol. Cancer Ther.

[143] Ohno S., Takanashi M., Sudo K., Ueda S., Ishikawa A., Matsuyama N., Fujita K., Mizutani T., Ohgi T., Ochiya T. (2013). Systemically injected exosomes targeted to EGFR deliver antitumor microRNA to breast cancer cells. Mol. Ther.

[144] Kooijmans S.A., Vader P., van Dommelen S.M., van Solinge W.W., Schiffelers R.M. (2012). Exosome mimetics: A novel class of drug delivery systems. Int. J. Nanomedicine.

